# Minimal Clinically Important Difference in the Six-Minute Walking Distance 12 Months After Lumbar Spinal Stenosis Surgery

**DOI:** 10.7759/cureus.81153

**Published:** 2025-03-25

**Authors:** Tomoyoshi Sakaguchi, Masato Tanaka, Jenil Patel, Bhaskar Sarkar, Meet Shah, Angel Oscar Paz Flores, Shinya Arataki, Tadashi Komatsubara, Yosuke Yasuda, Kazuhiro Miyata

**Affiliations:** 1 Department of Rehabilitation, Okayama Rosai Hospital, Okayama, JPN; 2 Department of Orthopedic Surgery, Okayama Rosai Hospital, Okayama, JPN; 3 Department of Physical Therapy, Ibaraki Prefectural University of Health Science, Ibaraki, JPN

**Keywords:** lumbar spinal stenosis, minimal clinically important difference, musculoskeletal rehabilitation, six-minute walk distance, spine surgery

## Abstract

Study design: This was a prospective single-center study.

Purpose: This study aimed to calculate the minimal clinically important difference (MCID) of the six-minute walk distance (6MWD) postoperatively.

Overview of the literature: The 6MWD and Zurich Claudication Questionnaire (ZCQ) have recently been used to evaluate the intermittent claudication of lumbar spinal stenosis (LSS). However, there is no report concerning the MCID of postoperative changes of 6MWD in patients with LSS.

Methods: This prospective study included patients aged 60 or older who underwent surgery for LSS with intermittent claudication as the primary symptom at a single institution from 2022 to 2024. The 6MWD and the ZCQ were assessed preoperatively and 12 months postoperatively. The MCID was evaluated using an anchor-based approach using ZCQ. The Spearman's rank correlation coefficient was used to determine the correlation between the pre-and postoperative changes in 6MWD and ZCQ. The MCID was calculated using the receiver operating characteristic (ROC) curve. Calculated MCID and area under the curve (AUC) were evaluated by internal validation using the bootstrap method. A significance level of <0.05 was used, and bootstrap validation was performed with a 95% confidence interval.

Results: One hundred and five patients were included in the study. The average preoperative and postoperative 6MWD were 282 meters and 385 meters, respectively (p<0.01). The ZCQ significantly improved from 2.64 to 1.73 postoperatively (p<0.01). Postoperative 6MWD and ZCQ correlated substantially with change (r=-0.58, p<0.01). In the anchor-based approach, 67 patients (63%) responded to the anchor ZCQ with an MCID of 80.0 m. This MCID had a sensitivity of 92.1%, a specificity of 76.1%, and an AUC of 90.6%, which was excellent. Internal validation showed moderate to excellent reliability with 95% confidence intervals for MCID and AUC ranging from 40 m to 100 m and 86% to 95%, respectively.

Conclusions: In this study, the MCID of postoperative changes of the 6MWD in patients with LSS was 80 m. This value helps evaluate the surgical results of patients with LSS.

## Introduction

Lumbar spinal stenosis (LSS) is the most common cause of lumbar spine surgery in elderly patients [1]. Lumbar spinal stenosis is characterized by pain in the buttocks or lower extremities, which may occur with or without low back pain, and is associated with a reduction in the space available for the neural and vascular structures of the lumbar spine [1]. The main symptom of LSS is intermittent claudication (IC) due to compression and ischemia to the nerve roots and cauda equina, with decreased ability to walk [2]. Intermittent claudication has a significant impact on physical activity and quality of life, and LSS patients expect surgery to improve their IC [3]. Surgery for LSS includes decompression and fusion surgery, which improves leg pain, leg numbness, and IC in LSS patients compared to non-surgical treatment.

To evaluate the severity of IC in LSS patients, selecting the candidate for surgery is very important. Self-reported walking distance underestimates the actual walking distance by 30% and is unreliable [4]. The motorized treadmill test, self-paced walking test, and self-reported walking distance have been used for objective assessment of IC [5]. However, motorized treadmill tests require up to 30 minutes for measurement and are difficult to use clinically [5]. The six-minute walk distance (6MWD) has recently been used to evaluate the IC of LSS [6]. Improvement in 6MWD is associated with treatment satisfaction in postoperative LSS patients [7]. Therefore, 6MWD is an essential measure of IC after LSS surgery. The Zurich Claudication Questionnaire (ZCQ) is used for patient-reported outcomes of IC [8]. The ZCQ is a disease-specific quality-of-life rating scale for LSS and is the most used worldwide. The ZCQ is the primary indicator that responds best to LSS surgery [9]. The ZCQ has also been reported to respond to changes in 6MWD after LSS surgery [7].

The minimal clinically important difference (MCID) is defined as “the smallest difference in a score that patients perceive as beneficial and that would lead, in the absence of harmful side effects or high costs, to adaptations in care management.” The MCID measures treatment efficacy in walking ability and balance function after spine disease surgery and intervention [10,11]. Although MCID is disease-specific, MCID for pulmonary and cardiac disease was used to interpret changes in 6MWD after previous LSS surgery [12]. To evaluate the improvement in IC for LSS, the MCID of 6MWD for LSS needs to be calculated. This study aimed to estimate the MCID of 6MWD 12 months after LSS surgery using the ZCQ as the anchor.

## Materials and methods

**Figure 1:**
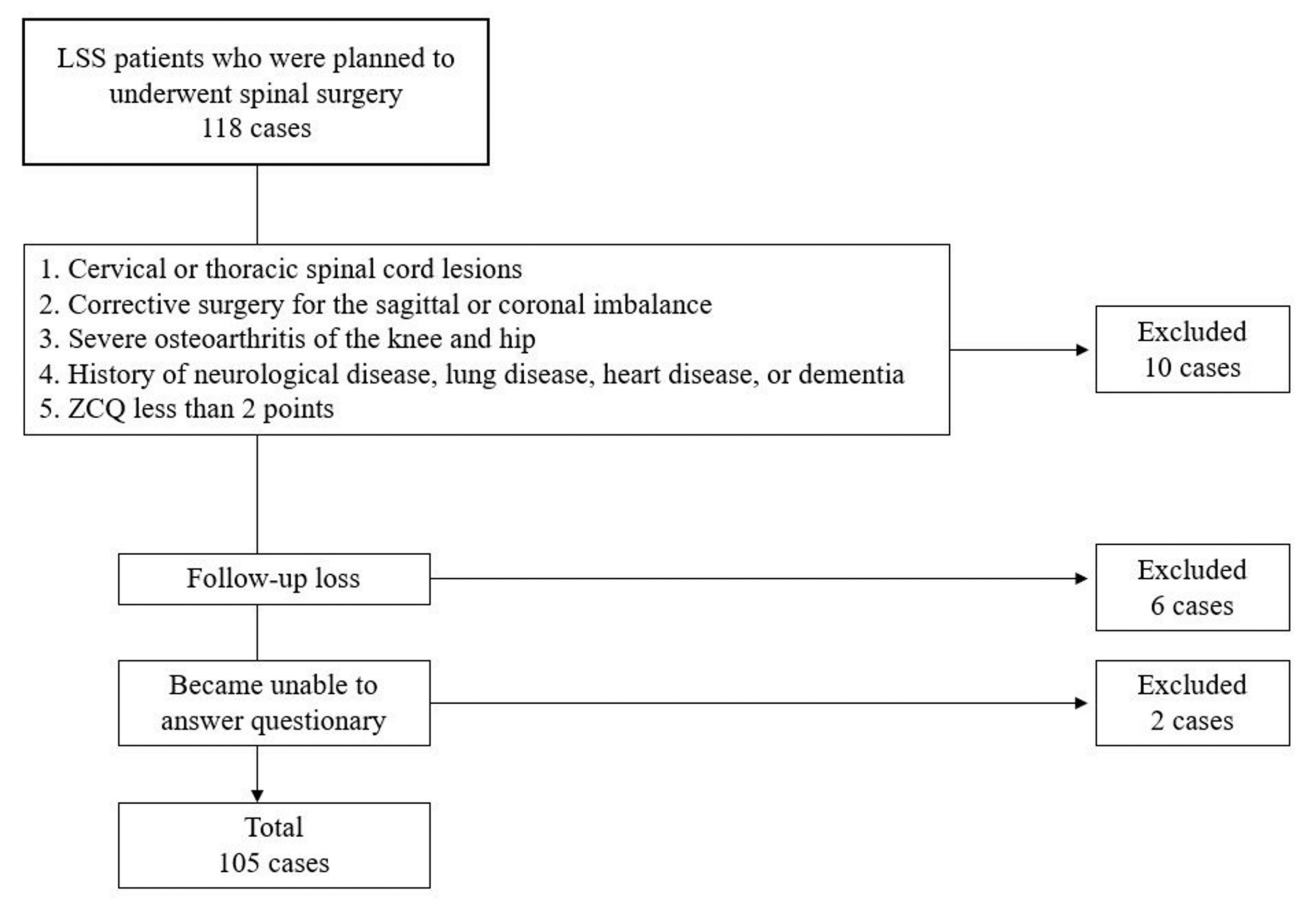
Patient selection flowchart LSS: lumbar spinal stenosis; ZCQ: Zurich Claudication Questionnaire

This is a single-center, prospective study. The institution's ethics committee of Okayama Rosai Hospital, Okayama, Japan has approved the study (approval no. 348-3). Patients were informed of the study's content, and their consent was obtained in writing. Consecutive patients who underwent surgery for LSS at Okayama Rosai Hospital from April 2022 to April 2024 were included in the study. The inclusion criteria were (1) age ≥60 years; (2) intermittent claudication due to leg pain and/or numbness; (3) results of the patient-reported outcome of ZCQ available; (4) informed consent available. The exclusion criteria were (1) cervical or thoracic spinal cord lesions; (2) corrective surgery for the sagittal or coronal imbalance; (3) severe osteoarthritis of the knee and hip; (4) history of neurological disease, lung disease, heart disease, or dementia; (5) ZCQ less than two points. A total of 105 patients were included in this study (Figure 1).

Outcome measures

The 6MWD was measured to assess IC, and the ZCQ was evaluated as the anchor to calculate MCID. These measures were assessed preoperatively and 12 months postoperatively to determine clinical significance.

Six-minute walking distance

The patients were instructed to walk for six minutes as far as possible on a flat path 30 meters long (Figure 1).

**Figure 2:**
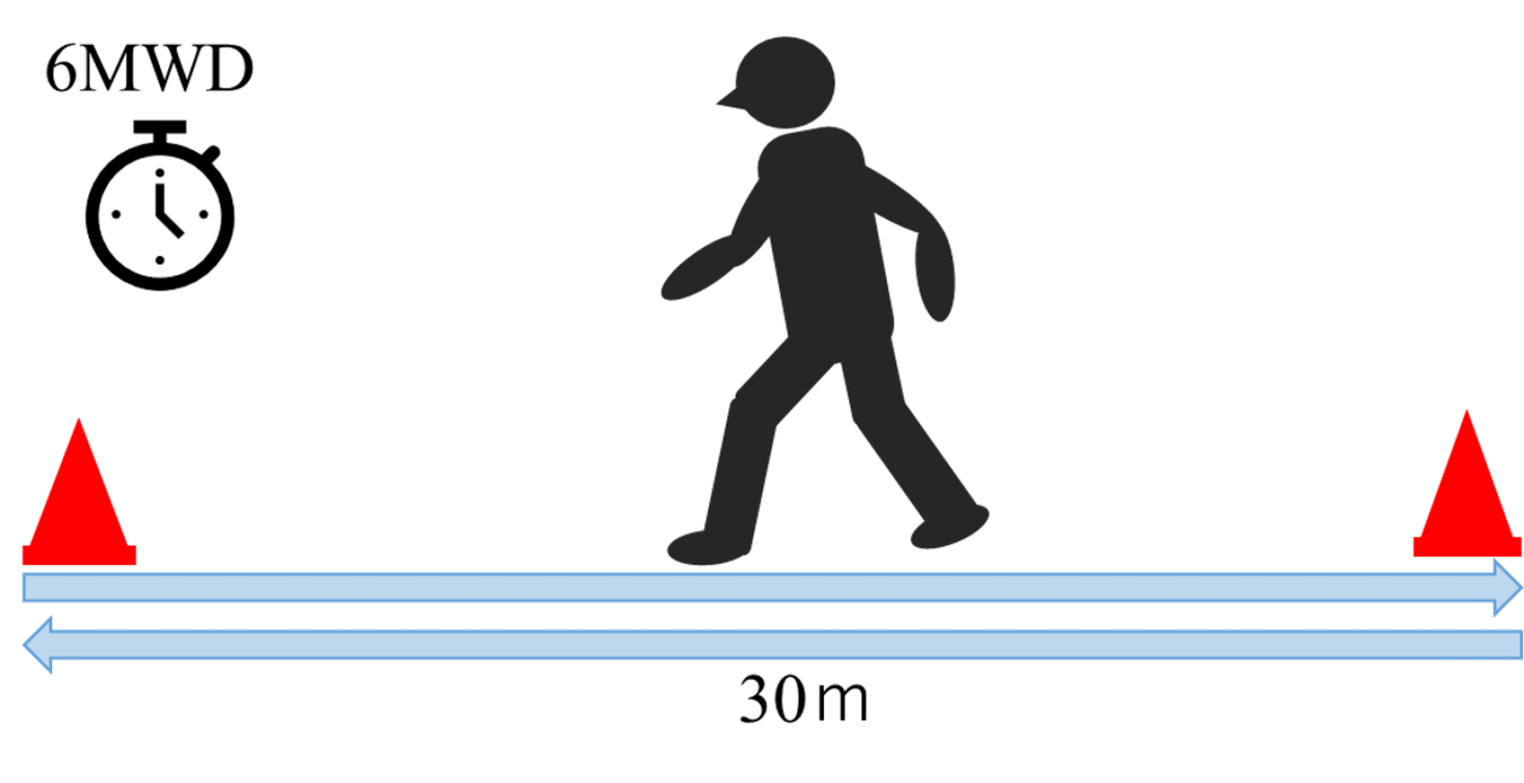
Six-minute walking distance (6MWD) Patients were instructed to walk for six minutes on a flat path which was 30 meters long. Image credit: Tomoyoshi Sakaguchi

The measured 6MWD was recorded as the total distance walked in six minutes. Measurements were taken by one examiner per examinee. The examiner walked with the patient, taking care to stay out of the patient's sight to control the risk of falling. The patients did not use walking aids whenever possible, and a cane was permitted only if they could not walk. The use of a walker was not allowed.

The ZCQ

The ZCQ is presented in Table 1.

**Table 1:** Physical Function Scale from Zurich Claudication Questionnaire Source: [8]

Physical Function Scale from Zurich Claudication Questionnaire
I. How far have you been able to walk? 1. More than 2 miles; 2. More than 2 blocks, but less than 2 miles; 3. More than 50 feet, but less than 2 miles; 4. Less than 50 feet
II. Have you taken walks outdoors or around the shops for pleasure? 1. Yes, comfortably; 2. Yes, but sometimes with pain; 3. Yes, but always with pain; 4. No
III. Have you been shopping for groceries or other items? 1. Yes, comfortably; 2. Yes, but sometimes with pain; 3. Yes, but always with pain; 4. No
IV. Have you walked around the different rooms in your house or apartment? 1. Yes, comfortably; 2. Yes, but sometimes with pain; 3. Yes, but always with pain; 4. No
V. Have you walked from your bedroom to the bathroom? 1. Yes, comfortably 2. Yes, but sometimes with pain 3. Yes, but always with pain 4. No

The ZCQ is a disease-specific outcome measure for LSS. It consists of three subscales: the Symptom Severity Scale, the Physical Function Scale (PFS), and the Patient Satisfaction Scale. The PFS specifically assesses limitations in daily activities due to IC caused by LSS. The MCID of the ZCQ PFS is reported to be 0.60 [13].

Statistical analysis

External validation of the previous MCID [14] was performed. Initially, changes in 6MWD and ZCQ before and after surgery and the correlation between 6MWD and ZCQ were evaluated; the MCID was calculated using the anchor-based approach. The calculated MCID was evaluated for robustness by internal validation. Statistical analysis was performed using EZR Ver. 1.64 (Division of Hematology, Saitama Medical Center, Jichi Medical University, Saitama, Japan)[15]) and R-4.3.2 (Hornik and R Core Team (2024), https://cran.r-project.org/bin/windows/base/). A p-value of <0.05 was considered statistically significant.

External Validation

To assess clinical utility, contingency tables were created using the MCID from the previous study, and sensitivity, specificity, and positive and negative likelihood ratios were calculated.

Anchor-Based Approach

The ZCQ is used to evaluate intermittent claudication. The main symptom of LSS patients is not low back pain but intermittent claudication due to leg pain or numbness, so we used the anchor as ZCQ.

The assessment of improvement in 6MWD and ZCQ preoperatively and 12 months postoperatively was calculated using the Wilcoxon signed rank test. ZCQ is used to evaluate intermittent claudication, and the main symptom of LSS patients is not low back pain but intermittent claudication due to leg pain or numbness.

Spearman's rank correlation coefficient was used to evaluate the correlation between 6MWD and the change in ZCQ preoperatively and 12 months postoperatively. The r is defined as 0.1 to <0.4 is weak, 0.4 to <0.7 is moderate, 0.7 to <0.9 is described as strong, and ≥0.9 is very strong [16].

Patients were classified into response and non-response groups using the MCID of 0.60 points for ZCQ as an anchor [13]. The receiver operating characteristic (ROC) curve was used to calculate MCID, sensitivity, specificity, and area under the curve (AUC) for 6MWD. The AUC thresholds were interpreted as follows: 90% to 100%: excellent discrimination, 80% to 90%: good, 70% to 80%: acceptable, 60% to 70%: poor, 50% to 60%: fail, and 50% indicating no discriminatory power [17].

Internal Validation

As internal validation, we calculated the 95% confidence interval of the newly calculated MCID and AUC using the bootstrap method.

## Results

Patient demographics

Preoperative evaluations were performed on 118 patients. Thirteen patients who met the exclusion criteria by the 12-month postoperative evaluation were excluded, and 105 were included in the study (62 men and 43 women). The mean age at surgery was 72.6 ± 8.4 years (Table 2).

**Table 2:** Patient demographics BMI: body mass Index; VAS: visual analogue scale; ZCQ: Zurich Claudication Questionnaire; 6MWD: six-minute walking distance

Parameters			Mean ± SD; n (%)	
	Total (n=105)	Achieved (n=67)	Not achieved (n=38)	p-value
Age (years)	72.6±8.4	71.6±8.2	74.2±8.4	0.12
Sex				0.63
Male	62 (59%)	41 (61%)	21 (55%)	
Female	43 (41%)	26 (39%)	17 (45%)	
BMI (kg/m²)	24.3±3.2	24.1±3.3	24.7±3.1	0.63
Surgical type				0.19
Decompression	64 (61%)	37 (55%)	27 (71%)	
Fusion	41 (39%)	30 (45%)	11 (29%)	
Surgical segments				0.09
1	73 (70%)	51 (76%)	22 (58%)	
≥2	32 (30%)	16 (24%)	16 (42%)	
Duration of symptoms (year)	1.4±2.1	1.9±1.5	2.4±3.5	0.09
Low back pain VAS (0–100)	43.8±30.4	46.4±30.9	39.4±29.2	0.26
Lower limb pain VAS (0–100)	51.3±31	55.1±31.6	44.8±29.2	0.14
Lower limb numbness VAS (0–100)	42.9±32.4	44.2±32.8	40.6±31.47	0.56
ZC	2.64±0.54	2.87±0.47	2.23±0.41	<0.01
Preoperative 6MWD (m)	281.8±113.8	249.4±110.2	338.1±97.2	<0.01

Comparing the previous and current subjects, the current subject had a shorter preoperative 6MWD and severely reduced walking ability.

External validation

The previous MCID and a contingency table are shown below (Table 3). The clinical usefulness evaluation showed a sensitivity of 81%, a specificity of 61%, a positive likelihood ratio of 2.04%, and a negative likelihood ratio of 0.32%, indicating that the previous MCID was not helpful in this cohort.

**Table 3:** Contingency table using previous MCID MCID: minimally clinically important difference; ZCQ: Zurich Claudication Questionnaire; 6MWD: six-minute walking distance

	MCID for ZCQ achieved	MCID for ZCQ not achieved	Total
MCID for 6MWD achieved	54	15	69
MCID for 6MWD not achieved	13	23	36
Total	67	38	105

Anchor-based approach

The 6MWD significantly improved from preoperative 282 ± 114 m to 385 ± 89.0 m (p<0.01). The mean change in 6MWD was 103 m. The ZCQ significantly improved from 2.64 ± 0.54 to 1.73 ± 0.57 (p<0.01). The mean change in ZCQ was -0.91. A significant correlation was found between the change in 6MWD and ZCQ preoperatively and 12 months postoperatively (r=-0.58, p<0.01) (Figure 2).

**Figure 3:**
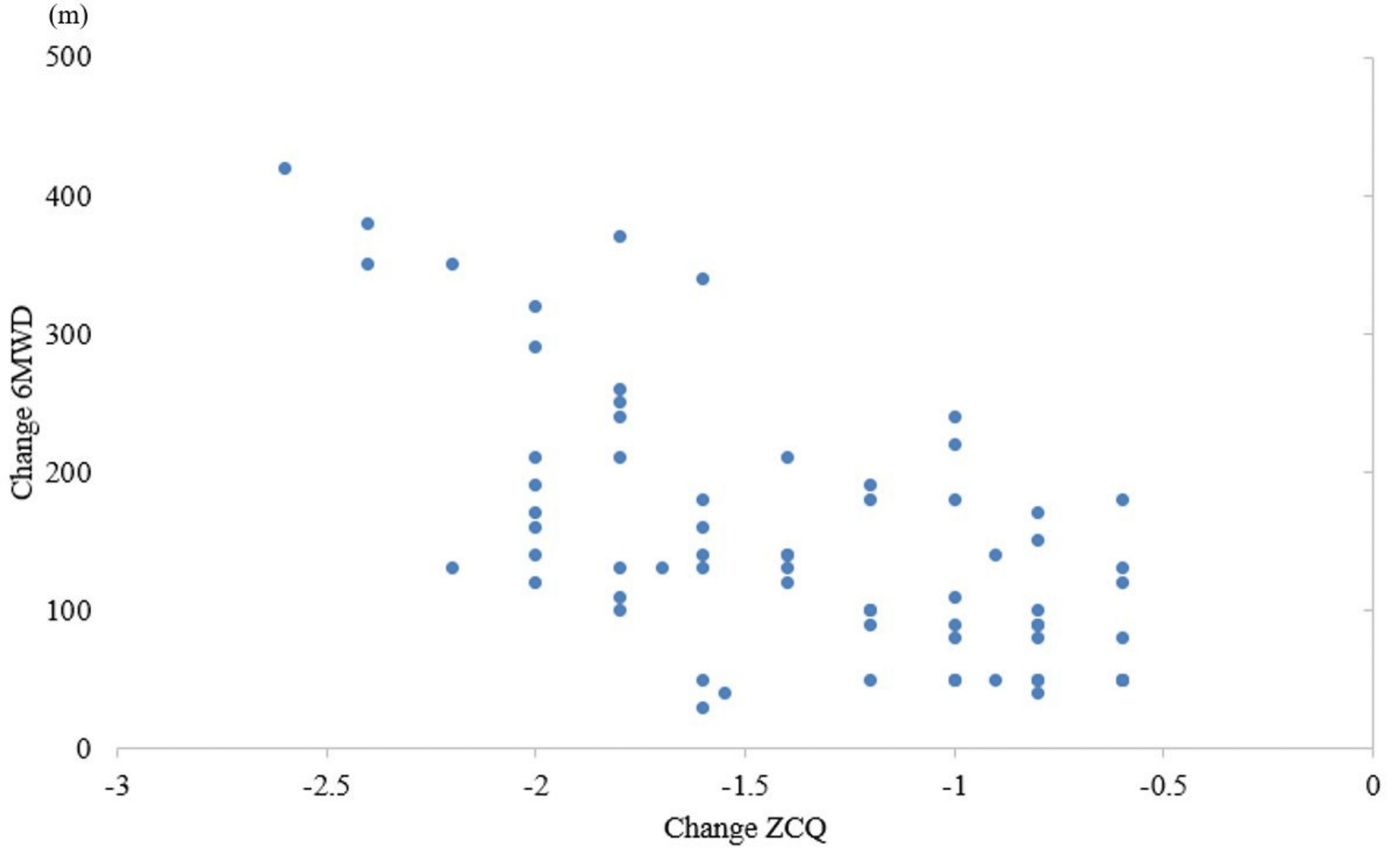
Correlation between 6MWD and change in ZCQ PFS ZCQ PFS: Zurich Claudication Questionnaire Physical Function Score; 6MWD: six-minute walking distance

Sixty-seven patients (63%) responded to MCID for ZCQ with Anchor. The ROC curve showed an MCID of 80 meters, and the 95% confidence interval for AUC was 85.2% to 96.1%, with a sensitivity of 92.1%, a specificity of 76.1%, and an AUC of 90.6%. (Figure 3).

**Figure 4:**
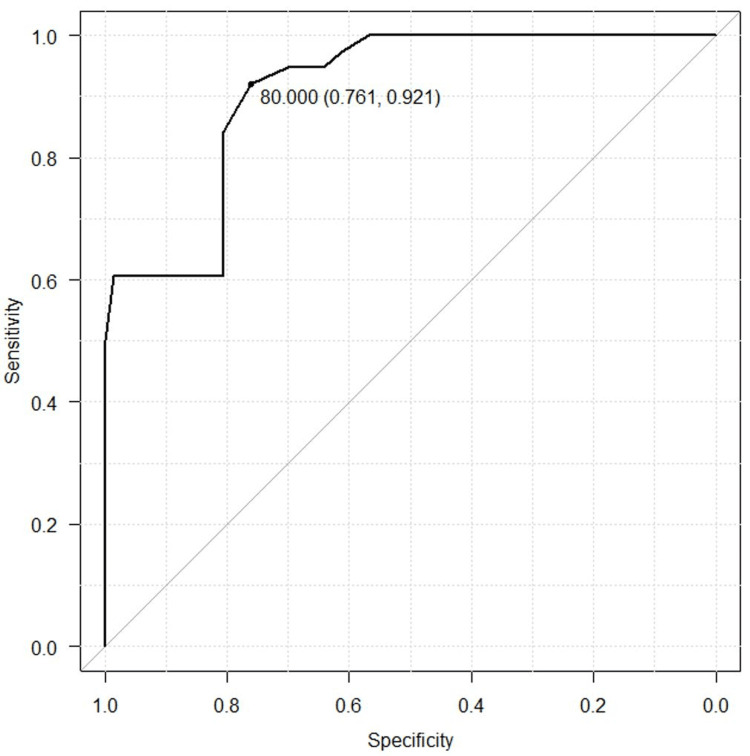
The ROC curve calculates the MCID of 6MWD ZCQ PFS: Zurich Claudication Questionnaire Physical Function Score; 6MWD: six-minute walking distance; ROC: receiver operating characteristic

Internal validation

Internal validation by the bootstrap method showed that the 95% confidence intervals for MCID and AUC were 40 meters to 100 meters and 86% to 95%, respectively.

## Discussion

Intermittent claudication is the most common clinical manifestation of LSS and significantly impairs the ability to walk due to back pain, leg pain, and leg weakness. Many reports show that surgery for LSS dramatically improved patients’ IC [18]. The 6MWD and ZCQ have recently been used to assess the IC in patients with LSS [6]. The ZCQ better reflects the surgery results for LSS than patient-reported outcomes such as the Oswestry Disability Index (ODI) and the eight-item short-form health survey [9].

In this study, the mean change in ZCQ 12 months after LSS surgery was -0.91, better than other reports (-0.58 to -0.76) [13, 19]. This difference in improved surgical results is because only spinal decompression was performed in the previously reported study, whereas both decompression and spinal fusion were performed in this study. Spinal fusion has been reported to improve leg pain and numbness in LSS compared to decompression [20]. The ZCQ consists of questions about average daily walking distance and indoor and outdoor walking (Table 1). Average scores are calculated and evaluated, with scores increasing with increasing severity. The ZCQ can also be considered a self-administered measure to assess symptom severity, physical function, and surgery satisfaction in LSS. This study's mean change in 6MWD from preoperative to 12 months postoperative was 103 m. Previous studies have reported mean changes in 6MWD 12 months after LSS surgery of 73 meters to 132 meters [14, 21], comparable to our result. The 6MWD relates to actual outdoor walking ability and health-related quality of life in patients with IC [22].

Our result was that the MCID of 6MWD 12 months after LSS surgery with ZCQ as the anchor was 80 meters. This study showed excellent discrimination performance compared to previous studies (sensitivity 72%, specificity 65%, and AUC 78%) [14, 17]. It has been reported that the 6MWD in LSS less than 311 meters severely declines walking ability [14]. Patients with a 6MWD of less than 300 meters are defined as frail [23]. The subjects in this study were LSS with a mean preoperative 6MWD of 282 meters and severe IC. Baseline severity can significantly influence the calculated MCID, as patients with lower baseline walking ability may require larger absolute improvements to perceive a meaningful clinical change. Previous studies have observed this phenomenon, emphasizing the importance of considering baseline characteristics when interpreting MCID results [24]. Our cohort study had more severe cases and required a more significant improvement value of 6MWD to obtain a clinically beneficial change than in previous studies [14]. Internal validation showed moderate to excellent reliability with 95% confidence intervals for MCID and AUC ranging from 40 meters to 100 meters and 86% to 95%, respectively.

Takenaka et al. reported that the MCID of 6MWD 12 months after LSS surgery was 57.5 meters [14], which is shorter than our result. The discrepancy can be attributed to several key differences. First, they used ODI as the anchor for the 6MWD. The ODI evaluates the activity of daily living due to low back pain and lack of walking ability. Therefore, more than ODI may be required as an anchor for calculating the MCID for 6MWD. Second, our cohort had a lower preoperative 6MWD (282 meters) compared to the previous study (311 meters), indicating a more severely impaired population. Patients with more severe functional limitations may require greater absolute improvements in walking ability to perceive a meaningful clinical change, leading to a higher MCID. Based on these findings, external validation was conducted to assess the utility of the MCID of 57.5 m for the 6MWD in evaluating clinical improvement at 12 months following LSS surgery. As a result, the previous MCID did not meet the criterion value for positive and negative likelihood ratios [25], and its usefulness could have been higher in our cohort.

The usefulness of an anchor-based approach depends mainly on the relationship between the measured outcome measure and the anchor [26]. Prior studies used the ODI [27] for the anchor, and we used the ZCQ [8]. The ZCQ used as an anchor in this study is an LSS-specific rating scale consisting solely of questions related to walking, such as subjective walking distance and indoor/outdoor mobility. The correlation coefficient with 6MWD was -0.58 when the ZCQ was used to anchor the external reactivity assessment. The moderate correlation between 6MWD and ZCQ supports using ZCQ as an anchor for calculating MCID [28]. When calculating MCID, the more responsive the outcome is to treatment and anchors, the more sensitive it is to changes in the patient's condition, and the more appropriate MCID [29].

Our study's correlation coefficient between 6MWD and ZCQ was r = -0.58, indicating a moderate inverse relationship. This means that as patients report improved symptoms and functional status on the ZCQ, their 6MWD tends to increase. For clinicians, this moderate correlation suggests that while 6MWD reflects improvements in walking ability, it is also influenced by other factors such as physical endurance and overall mobility. The use of ZCQ as an anchor is particularly relevant because it assesses not only walking ability but also the impact of these limitations on the quality of life in LSS patients. This makes the calculated MCID of 80 m a meaningful threshold for evaluating postoperative recovery.

The MCID of 80 meters calculated in this study can serve as a benchmark for evaluating the improvement of 6MWD in LSS patients. Clinicians can use this threshold to identify patients likely to benefit most from intervention and tailor rehabilitation strategies to achieve this clinically significant improvement. The calculated MCID is used to clinically interpret postoperative results and research to achieve MCID [30]. These 80 meters can be used as a criterion to investigate factors to achieve MCID of 6MWD in postoperative LSS patients.

This study has several limitations. First, its single-center scope may introduce selection bias, limiting the generalizability of the findings to broader patient populations. Thus, caution is needed when generalizing the findings to other institutions or regions, and the generalizability to wider patient populations remains uncertain. Second, the relatively small sample size further restricts the robustness of the conclusions. Third, the follow-up period was limited to 12 months postoperatively, preventing an assessment of long-term surgical outcomes and recovery patterns over time. Additionally, potential biases such as loss to follow-up could have influenced the results. Fourth, the evaluation of surgical outcomes relied solely on the ZCQ without incorporating other patient-reported measures, such as the global change scale, which may have provided a more comprehensive assessment. Future studies with larger, multicenter cohorts, extended follow-up periods, and diverse assessment tools are necessary to validate and refine these findings.

## Conclusions

This study demonstrates that 6MWD significantly improves 12 months following LSS surgery, with a mean change of 104 meters. The MCID for 6MWD at 12 months postoperatively, with ZCQ as the anchor, was 80 meters. This MCID provides a meaningful threshold for assessing clinically significant improvements in walking ability. The high sensitivity (92.1%) and specificity (76.1%) suggest that this threshold helps identify patients who show meaningful improvements. The results underline the importance of using the ZCQ as an anchor, reflecting the specific walking limitations and quality of life issues in LSS patients. Future research with larger cohorts and longer follow-ups will help validate and expand these findings.
